# Differential Accumulation of sHSPs Isoforms during Desiccation of the Resurrection Plant *Haberlea rhodopensis* Friv. under Optimal and High Temperature

**DOI:** 10.3390/life13010238

**Published:** 2023-01-14

**Authors:** Gergana Mihailova, Magdalena Tchorbadjieva, Goritsa Rakleova, Katya Georgieva

**Affiliations:** 1 Institute of Plant Physiology and Genetics, Bulgarian Academy of Sciences, Acad. G. Bonchev Str., Bl. 21, 1113 Sofia, Bulgaria; 2 Department of Biochemistry, Faculty of Biology, Sofia University, 8 Dragan Tsankov Blvd., 1164 Sofia, Bulgaria

**Keywords:** drought stress, high temperature stress, desiccation tolerance, chlorophyll fluorescence, non-photochemical quenching, 1D and 2D SDS-PAGE, Western blot, small heat shock proteins

## Abstract

**Simple Summary:**

*Haberlea rhodopensis* (Gesneriaceae) belongs to the group of so-called resurrection plants, which are able to lose more than 95% of the water in the cells and quickly restore their metabolism upon rehydration. The plant is characterized with a high ecological plasticity growing at altitude from 136 m to near 1700 m at different temperature, water, and light conditions. In its natural habitats, *H. rhodopensis* is often exposed to high temperatures during dry periods in the summer. In the present study, we investigated the accumulation of small heat shock proteins (sHSPs) and the extent of non-photochemical quenching during the downregulation of photosynthesis in *H. rhodopensis* under desiccation at optimum (23 °C) and high temperature (38 °C). Dehydration at high temperature has a detrimental effect on plant photosynthesis, thus leading to oxidative stress and damage of different important macromolecules in the cells. Plants accumulate different protective proteins when exposed to high temperatures, one of the most characteristic being sHSPs. They prevent irreversible aggregation of unfolded or aggregated proteins and facilitate their refolding. Plants possess more than 30 different of sHSPs, much more compared to mammals and microorganisms. Fourteen different isoforms of sHSPs accumulate during dehydration at 38 °C compared to 23 °C, where only two were detected. *H. rhodopensis* is an excellent model system to study the protective mechanisms under extreme dehydration at high temperature, thus helping plant breeders to create high temperature resistant crops.

**Abstract:**

*Haberlea rhodopensis* belongs to the small group of angiosperms that can survive desiccation to air-dry state and quickly restore their metabolism upon rehydration. In the present study, we investigated the accumulation of sHSPs and the extent of non-photochemical quenching during the downregulation of photosynthesis in *H. rhodopensis* leaves under desiccation at optimum (23 °C) and high temperature (38 °C). Desiccation of plants at 38 °C caused a stronger reduction in photosynthetic activity and corresponding enhancement in thermal energy dissipation. The accumulation of sHSPs was investigated by Western blot. While no expression of sHPSs was detected in the unstressed control sample, exposure of well-hydrated plants to high temperature induced an accumulation of sHSPs. Only a faint signal was observed at 50% RWC when dehydration was applied at 23 °C. Several cross-reacting polypeptide bands in the range of 16.5–19 kDa were observed in plants desiccated at high temperature. Two-dimensional electrophoresis and immunoblotting revealed the presence of several sHSPs with close molecular masses and pIs in the range of 5–8.0 that differed for each stage of treatment. At the latest stages of desiccation, fourteen different sHSPs could be distinguished, indicating that sHSPs might play a crucial role in *H. rhodopensis* under dehydration at high temperatures.

## 1. Introduction

Resurrection plants are a small group of angiosperms that can tolerate desiccation to air dry state and quickly resume their normal physiological functions upon rehydration [1]. *Haberlea rhodopensis* is a homoiochlorophyllous resurrection plant, since it retains its chlorophyll content during dehydration [2]. This shade plant is a Tertiary relict that emerged in the late Oligocene [3] and a Balkan endemic from the family Gesneriaceae. *H. rhodopensis* is characterized with a high ecological plasticity, growing at altitudes from 136 m to nearly 1700 m at different temperature, water, and light conditions [4]. Since the plant retains its chlorophyll content and maintains its photosynthetic apparatus during dehydration, this enhances a risk of excessive production of harmful reactive oxygen species (ROS) that can induce oxidative stress in the plant cells [5]. ROS can cause damage to subcellular compartments and macromolecules, e.g., lipids, proteins, and nucleic acids [6]. To minimize ROS production and free radical-caused damage during drying, resurrection plants use different mechanisms: downregulation of metabolism/photosynthesis, production of sugars and various compatible solutes, and accumulation of antioxidants and proteins, such as the late embryogenesis abundant proteins (LEA), including dehydrins and heat shock proteins (HSPs) [7].

Under natural conditions dehydration of the *H. rhodopensis* is often accompanied by low or high temperature and/or high light intensities [4]. Our previous investigations showed that dehydration at high temperature reduced to a greater extent the photosynthetic rate, photochemical activity of PSII and PSI, and the amount of the main PSI and PSII proteins and led to higher levels of distribution of excitation energy to PSI, dark respiration, alternative electron sinks, and catalase activity compared to dehydration at optimal temperature [8,9,10,11,12]. High temperature stress affects the metabolism and cellular organization of plants, and can cause protein denaturation, aggregation or degradation, enzyme deactivation, inhibition of protein synthesis and loss of membrane integrity [13,14]. The expression of stress proteins, such as HSPs, LEA proteins, including dehydrins, cytosolic CuZn-SOD and Mn-peroxidase, and ubiquitin, plays an important role in protecting cell structures from oxidative damage and high-temperature-induced dehydration [15,16]. High temperature exposure and signal perception alters gene expression, transcript accumulation and protein synthesis in the cells [17]. Such a group of proteins, playing a very important role and accumulating in response to heat stress, is the HSPs [14]. HSPs are highly conserved proteins, acting as molecular chaperons. They are able to bind an equal weight of substrate proteins and prevent the irreversible aggregation of unfolded or aggregated proteins and facilitate their refolding by the ATP-dependent Hsp70/DnaK chaperones [18,19]. HSPs can be divided into six classes based on their molecular weight: HSP100, HSP90, HSP70, HSP60, HSP40/DNAJ family, and small HSPs (sHSPs) [20,21]. The most interesting group of HSPs are that of the sHSPs. Plants have a wide and diverse group of sHSPs compared to mammals and microorganisms [22], some plants possessing more than 30 different sHSPs. They are small proteins, with molecular mass ranging from 12 to 42 kDa (Sun et al., 2016), most in the range of 15–22 kDa [19]. Like all HSPs, sHSPs have a conservative C-terminally located 90 amino acid domain called the α-crystalline domain or heat shock domain flanked by an N- and a C-terminal region, with most sHSPs occurring in the form of oligomers [20,23,24]. Plant sHSPs are encoded by nuclear genes and can be divided into subfamilies, localized in different cell compartments: in cytosol or nucleus (CI-CVI), in mitochondria (MI-MII), and others in plastids (P), endoplasmic reticulum (ER), and peroxisomes (Po) [25,26]. Their large amount and heterogeneity suggest that they have unique physiological functions [27]. Some of sHSPs are constitutively expressed, being synthesized to a greater extent under high temperature [25]. In addition, plant sHSPs are expressed in response to a wide variety of stresses, including cold, drought, oxidative stress, heavy metals, injury, or during seed development and somatic embryogenesis [25,28,29].

Up to date, a few studies on the expression of sHSPs in resurrection plants are available in the literature. They are mainly related to transcriptomic studies [30,31,32,33,34] and only one has been performed at the protein level [35].

The present work aimed to investigate the expression of sHSPs in *Haberlea rhodopensis* leaves during desiccation at optimum and high temperature using electrophoretic and immunoblot assays. In addition, changes in the photosynthetic efficiency and non-photochemical chlorophyll fluorescence quenching under these conditions were compared. The interaction of water stress and high temperature is not well understood, especially in resurrection plants, and more investigations are still required.

## 2. Materials and Methods

### 2.1. Plant Material, Desiccation and Rehydration

Well-hydrated *Haberlea rhodopensis* plants were collected from their natural habitat where they grow on rocks below trees under very low irradiance. Adult rosettes from the same locality and of similar size and appearance were selected for the experiments. The tufts with naturally occurring thin soil layers were planted in pots in peat-soil. Plants were subjected to drought stress by withholding irrigation either at 23/20 °C or 38/30 °C day/night temperature, irradiance of 30 µmol m^−2^ s^–1^, 12 h photoperiod, and relative humidity of 60%. After desiccation to air-dry state, the plants were rehydrated in a modified desiccator, where the desiccant at the bottom was replaced by water, providing permanent humidity by a water pump. Control plants kept at 23/20 °C or 38/30 °C were regularly watered during the experiment. The samples were taken from well-hydrated control plants (95% RWC, C—control at 23 °C; 85% RWC, C3d, C7d—controls at 38 °C), moderately (RWC of 70% and 50%) and strongly (20% RWC) dehydrated, and dried leaves (8% RWC) as well as after 1 day (R1) and 7 days (R7) of rehydration of the dry plants at 23 °C and 38 °C.

### 2.2. Determination of Relative Water Content (RWC)

The RWC of leaves was determined gravimetrically by weighing them before and after oven drying at 80 °C to a constant mass and expressed as percentage of water content in dehydrated tissue compared to water-saturated tissues, using the equation: RWC (%) = (FM − DM)/(TM − DM) × 100, where FM denotes fresh mass, DM dry mass, and TM turgid mass. TM was measured on leaves maintained for 12–16 h at 4 °C in the dark floating on water.

### 2.3. Chlorophyll Fluorescence

Chlorophyll fluorescence emission was measured with a pulse amplitude modulation fluorometer (PAM 101-103, Walz, Effeltrich, Germany) according to Schreiber et al. [36]. Induction kinetics were registered and analyzed with the program FIP 4.3, written by Tyystjärvi and Karunen [37]. The maximum fluorescence yield Fm in the dark-adapted state and F_m_’ in the light-adapted state were measured by applying a 0.7 s pulse of white light (PPFD of 3500 µmol m^−2^ s^−1^, light source: KL 1500 electronic, Schott, Mainz, Germany). For the quenching analysis actinic white light (PPFD of 100 µmol m^−2^ s^−1^, KL 1500 electronic) was provided. F_o_′ was measured by turning off the actinic light and applying 3 s of weak far-red light (102-FR, Walz, emission peak at 730 nm). The ratio of the chlorophyll fluorescence decrease to steady-state fluorescence (used as a vitality index) was calculated as R_Fd_ = F_d_/F_s_, where F_d_ = F_m_ − F_s_ [38]. The non-photochemical quenching was calculated as qN = (F_v_ − F_v_′)/F_v_ [39].

### 2.4. Protein Extraction and One-Dimensional Sodium Dodecyl Sulphate Polyacrylamide Gel Electrophoresis (1D SDS-PAGE)

Total leaf proteins were extracted by incubating ground material in Laemmli buffer (62.5 mM Tris-HCl, pH 6.8, 10% glycerol, 2% SDS, and 5% β-mercaptoethanol) for 30 min at RT. After centrifugation for 10 min at 13,000× *g*, the supernatants were collected and the protein concentration was determined using a bicinchoninic acid kit (BCA 1, Sigma, Sigma-Aldrich, St. Louis, MO, USA). The protein samples were separated on 12% SDS-PAGE according to Okadjima et al. [40] using vertical electrophoresis unit SE 250 Mighty Small (Hoefer, San Francisco, CA, USA). Following SDS-PAGE, the gels were stained with 0.006% (*w*/*v*) Coomassie Brilliant Blue (CBB) R-250 in 50% (*v*/*v*) CH_3_OH, 10% (*v*/*v*) CH_3_COOH), or blotted on membrane. As molecular mass standards, prestained Precision Plus Protein^TM^ Dual Color Standards (Bio-Rad, Hercules, CA, USA) were used.

### 2.5. Two-Dimensional Gel Electrophoresis (2-DE)

To remove contaminants and to concentrate protein samples for isoelectric focusing (IEF) and 2D gel electrophoresis, the supernatants obtained as described in Section 2.4 were selectively precipitated using ReadyPrep™ 2-D Cleanup Kit (Bio-Rad, Hercules, CA, USA) according to the manufacturer’s instructions. The final protein pellet was dissolved in IEF lysis buffer (8M urea, 2% CHAPS, 0.5% Pharmalyte 2.5/8 (Amersham Pharmacia Biotech, Piscataway, NJ, USA), 40 mM DTT, 0.002% Bromphenol Blue). For 2-DE, proteins were resolved employing IEF on vertical 5% (*w*/*v*) polyacrylamide mini-slab gels under denaturing conditions in the presence of 8M urea [41]. IEF was carried out over a 2.5–8 pH gradient obtained by mixing 2.5–5.0 and 5–8 Pharmalyte ampholytes in a volume ratio 1.5:1. Following IEF, the gel was fixed, stained with CBB R-250, and the lane of interest was excised and equilibrated in SDS sample buffer for 15 min [41]. Next, the gel slice was placed on SDS stacking gel and the proteins were separated in 14% SDS-PAGE using the Hoefer SE260 mini vertical unit. Proteins in gels were visualized with 0.1% colloidal CBB G-250 or transferred on nitrocellulose membrane. Broad pI kit pH 3.5–9.3 (Amersham Pharmacia Biotech, Piscataway, NJ, USA) was used to determine the protein isoelectric points (pIs). As molecular mass standard, Fermentas PageRuler™ Unstained Protein Ladder (Thermo Fisher Scientific, Wilmington, DE, USA) was used.

### 2.6. Immunoblot Analysis

Using semi-dry transfer (TE70X, Hoefer, San Francisco, CA, USA), the proteins were blotted on nitrocellulose membrane for 50 min at a current of 0.8 mA per cm^2^. For evaluating the efficiency of protein transfer after SDS-PAGE, MemCode Reversible Protein Stain Kit was used (Pierce, Thermo Fisher Scientific, Wilmington, DE, USA). Blots were probed with primary antibodies against the α-crystalline domain of sHsp, kindly provided by Scott A. Heckathorn, University of Toledo (OH, USA). Anti-rabbit horseradish peroxidase-conjugated IgG antibody was used as secondary antibody. The protein bands were visualized by color reaction (6 mg DAB in 10 mL 50 mM (*w*/*v*) Tris-HCl, pH 7.6, 10 μL 30% H_2_O_2_).

### 2.7. Statistical Analysis

Dehydration of plants at 23 °C and 38 °C was repeated six times, each in three replications with standard error (*n* = 18). Isolation of total leaf proteins was performed twice, with samples taken from leaves of three different tufts for each treatment. Changes in the investigated parameters between plants desiccated at 38 °C and 23 °C were statistically compared by the Fisher least significant difference test at *p* ≤ 0.05 following ANOVA. A statistical software package (Statgraphics Plus, version 5.1 for Windows, The Plains, VA, USA) was used.

## 3. Results

### 3.1. Dehydration-Rehydration Cycle of Haberlea rhodopensis

**Figure 1:**
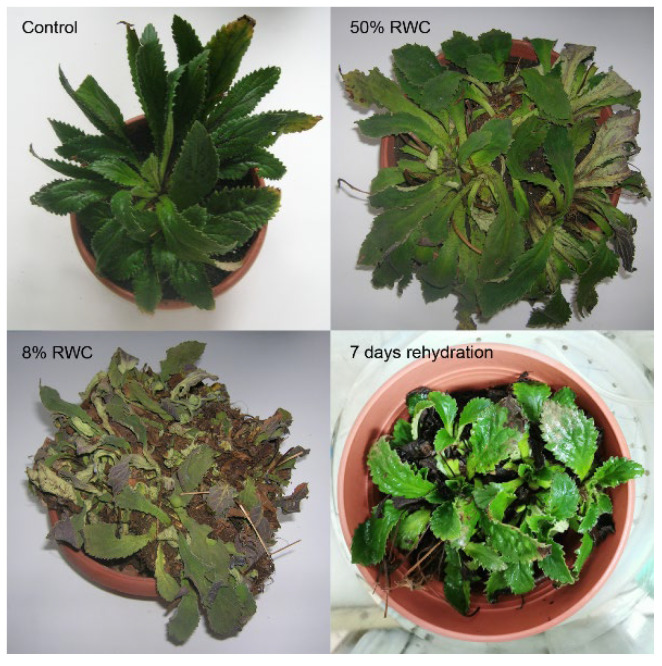
*H. rhodopensis* phenotype during dehydration-rehydration cycle.

The rate of water loss was three times higher in *H. rhodopensis* plants dehydrated at 38 °C compared to 23 °C. Plants reached air-dried state (8% RWC) for seven and 24 days under dehydration at 38 °C and 23 °C, respectively. During the experiments, leaves of control plants kept at 23 °C (C) did not change their RWC, while those which were regularly watered but exposed to high temperature reached 85% RWC after three days at 38 °C that did not change further till the end of the experiment. Rehydration of dried plants at 23 °C and 38 °C resulted in a rapid recovery of leaf RWC in one day (R1), i.e., 80% and 60%, respectively, and reached 95% after seven days of rehydration (R7). The *H. rhodopensis* phenotype during the dehydration–rehydration cycle is presented in Figure 1.

### 3.2. Changes in the Fluorescence Decrease Ratio (R_Fd_, Vitality Index) and the Extent of Non-Photochemical Quenching under Dehydration at 23 °C and 38 °C

**Figure 2:**
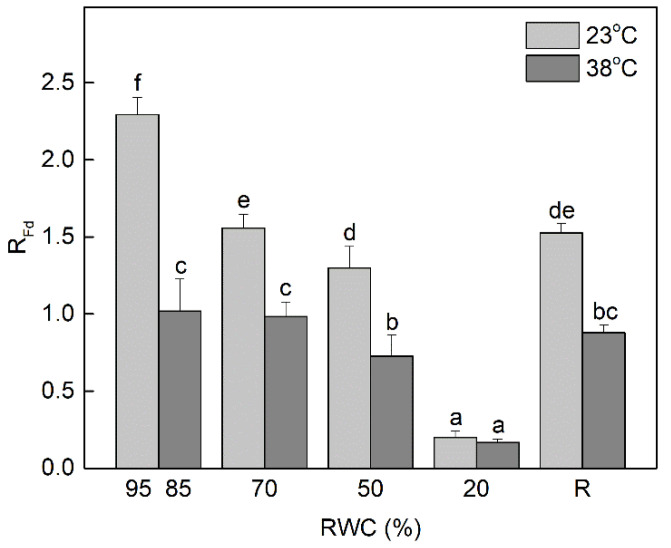
Changes of fluorescence decrease ratio (R_Fd_) in the controls (95, 85% RWC), during dehydration (70, 50, and 20% RWC) and after 7 days of rehydration (R7) of *H. rhodopensis* plants at optimal (23 °C) and high (38 °C) temperature. Changes between plants dehydrated at 23 °C and 38 °C were statistically compared. Data represent the mean of *n* = 18 with ±SE. Different letters within a graph indicate significant differences assessed by the Fisher LSD test (*p* ≤ 0.05) after performing multifactor ANOVA.

The ratio of Chl fluorescence decrease (F_m_ − F_s_) to the steady-state Chl fluorescence (F_s_), R_Fd_ permits a fast evaluation of the photosynthetic activity and vitality of plants under stress conditions. It has been shown that the changes in R_Fd_ values are in good agreement with CO_2_ assimilation rate of leaves [38]. Dehydration of *H. rhodopensis* at optimal temperature of 23 °C significantly decreased R_Fd_ values even at mild water deficit (70% RWC; *p* ≤ 0.05) (Figure 2). Further dehydration up to 50% RWC led to about 50% reduction of R_Fd_ and it was almost completely inhibited in severely desiccated plants (20% RWC). The R_Fd_ values were not completely recovered after seven days of rehydration of dry plants, they were approximately 30% lower compared to the well-hydrated control plants. Exposure of well hydrated plants to 38 °C slightly declined their RWC to 85% but resulted in more than 50% reduction of R_Fd_ values. Desiccation of plants at high temperature caused a stronger reduction in R_Fd_ than desiccation at optimal temperature. Indeed, the photosynthetic activity was almost completely inhibited in severely desiccated plants (20% RWC), regardless of the temperature.

**Figure 3:**
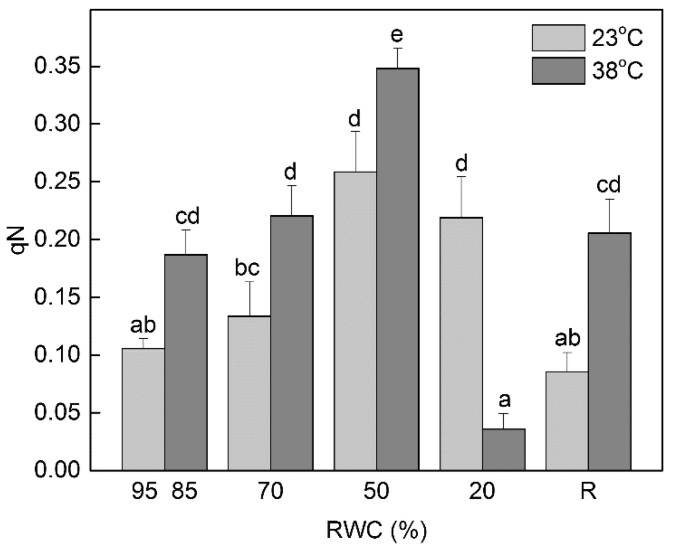
Changes in non-photochemical quenching (qN) in control plants (95, 85% RWC), during dehydration (70, 50, and 20% RWC) and after 7 days of rehydration (R7) of *H. rhodopensis* at optimal (23 °C) and high (38 °C) temperature. Changes between plants dehydrated at 23°C and 38°C were statistically compared. Data represent the mean of *n* = 18 with ±SE. Different letters within a graph indicate significant differences assessed by the Fisher LSD test (*p* ≤ 0.05) after performing multifactor ANOVA.

The decreased photosynthetic activity upon desiccation of plants at both optimal and high temperature was accompanied by an increase in non-photochemical quenching (qN) of Chl fluorescence (Figure 3). The values of qN reached maximum at 50% RWC, indicating the protective role of thermal energy dissipation under these conditions. Then, qN declined in severely dehydrated plants (20% RWC) and it was much stronger when it was performed at high temperature. Exposure of well-hydrated plants to 38 °C increased qN by about 80% compared to control plants kept at 23 °C. The values of qN were higher during desiccation at 38 °C up to 50% RWC than desiccation at 23 °C, as well as after rehydration.

### 3.3. Differential Accumulation of sHSPs during Dehydration at 23 °C and 38 °C

#### 3.3.1. One-Dimensional (1D) SDS-PAGE and Western Blots

**Figure 4:**
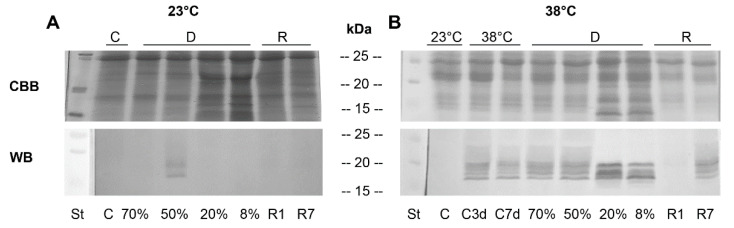
Analysis of sHSPs after 12% SDS-PAGE and Coomassie brilliant blue staining (CBB), coupled with Western blot (WB) using anti-sHSP antibody of *H. rhodopensis* control plants (C, C3d, C7d), dehydrated (70, 50, 20, and 8% RWC) and rehydrated after 1 and 7 days (R1 and R7, respectively). (**A**) 23 °C. (**B**) 38 °C. Each lane contains 30 µg soluble protein. Molecular mass markers are indicated at the left. St: Precision Plus Protein^TM^ Dual Color Standards (Bio-Rad, Hercules, CA, USA).

The sHSPs accumulation in *H. rhodopensis* plants during dehydration to air-dry state under optimal (23 °C) or high (38 °C) temperature and after rehydration was compared. To study the putative protective role of sHSPs during desiccation at 23 °C and 38 °C, we monitored the sHSPs protein expression by Western blot using antibodies raised against sHSPs. When the protein patterns after SDS-PAGE separation and CBB staining were compared, the most striking differences observed were at the late stages of dehydration both at optimal and high temperature (20% and 8% RWC) when new proteins in the molecular mass range of 10–30 kDa appeared (Figure 4A). The immunoblot analysis revealed only a faint signal at 50% RWC during desiccation at 23 °C distinguishing two bands of 17 and 19 kDa. However, when the plants were desiccated at high temperature (38 °C), the immunoblot results showed the presence of at least six different bands of apparent molecular weight 15–20 kDa in all investigated samples, except for the control (C) (Figure 4B). Only a very faint band could be seen in samples after one day rehydration (R1) (Figure 4B). The accumulation pattern changed both quantitatively and qualitatively during the course of desiccation, showing that sHSPs were differentially expressed in response to desiccation at high temperature. In addition, a different pattern of sHSPs expression was observed after full rehydration of plants (R7).

#### 3.3.2. 2D SDS-PAGE and Western Blots

**Figure 5:**
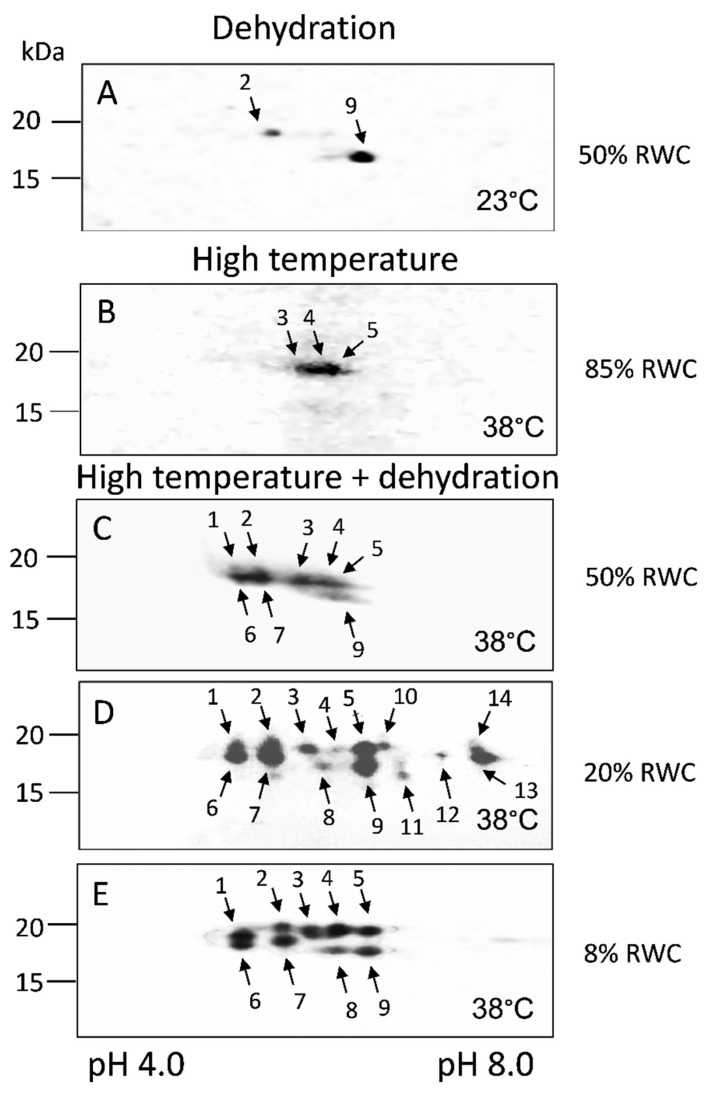
2-D Western blot analysis of sHSPs in *H. rhodopensis*. Total leaf proteins (50 μg) extracted from plants dehydrated at optimal temperature ((**A**), 23 °C; 50% RWC), control plants kept at 38 °C for three days ((**B**), C3d; 85% RWC) and plants dehydrated at high temperature ((**C**–**E**), 38 °C), were separated by 2-DE. The gels were transferred to nitrocellulose membrane and probed with anti-sHSP antibody at 1:1000 dilution. The proteins with equal mol. mass and pI are marked with the same number. Molecular mass markers are indicated at the left. St: Fermentas PageRuler™ Unstained Protein Ladder (Thermo Fisher Scientific, Wilmington, DE, USA).

For a more detailed analysis of the qualitative changes in the expression of sHSPs during desiccation at 23 °C and 38 °C, two-dimensional electrophoresis was performed (Figure 5). Immunoblotting after 2D electrophoresis revealed the presence of several sHSPs with close molecular weights (16.5–19 kDa) and pIs in the range of 5.0–8.0 (Figure 5).

The results showed that the two sHSPs detected after 1D electrophoresis and Western blot upon dehydration of *H. rhodopensis* to 50% RWC at 23 °C (Figure 4A), besides by molecular weight, differed by isoelectric point, too (no. 2 and no.9) (Figure 5A).

Three different sHSP isoforms were registered in control plants kept at 38 °C for three days (Figure 5B, C3d, no.3–no.5). An increase in the abundance of sHSP isoforms during the dehydration of plants at 38 °C was observed (Figure 5). Dehydration at high temperature enhanced sHSPs accumulation: eight isoforms were present at moderate dehydration (Figure 5C, no.1–no.7, no.9), fourteen in severely dehydrated (Figure 5D, no.1–no.14) and nine in air-dried plants (Figure 5E, no.1–no.9). Five isoforms were specific only for 20% RWC (Figure 5D, no.10–no.14) and one isoform for the latest stages of desiccation (Figure 5D,E, no. 8). From our results, we can conclude that different sHSPs isoforms in *H. rhodopensis* leaves accumulated in response to dehydration, high temperature, or dehydration at high temperature (Table 1). The molecular weight of the registered proteins is presented in Table 2.

## 4. Discussion

### 4.1. Photoprotective Mechanisms under Dehydration at 23 °C and 38 °C

In this study, we revealed some of the protective mechanisms on which resurrection species *H. rhodopensis* relied to overcome the detrimental effect of dehydration at high temperature. Desiccation of plants at high temperature induced a stronger decline of the vitality index (R_Fd_) compared to desiccation at optimal temperature and their recovery was slower. Thus, our results confirm that R_Fd_ is one of the most sensitive Chl fluorescence parameters. This ratio covers the whole process of photosynthesis, including the full induction period, the transition of the photosynthetic apparatus from the non-functional state 1 to its functional state 2, and the photosynthetic CO_2_ fixation [38]. A comparison of the changes in R_Fd_ and the rate of CO_2_ fixation during desiccation of *H. rhodopensis* [42] confirms the linear correlation between them as previously demonstrated by Lichtenthaler and Babani [43]. As might be expected, the decreased photosynthetic activity (R_Fd_) correlated with the enhanced values of non-photochemical quenching under moderate dehydration, especially at high temperature. The increased thermal dissipation of the excitation energy under stress is an important protective mechanism [44] by which the activity of PSII is downregulated, thus maintaining the balance between the photosynthetic electronic transport and carbon metabolism [45]. Elevated non-photochemical quenching under moderate water deficit is found also in *Ramonda serbica* and *Paraboea rufescens* [46,47]. It plays an important role in preventing photoinhibition in the initial stages of dehydration when electron transport and a proton gradient are still present, whereas at very low leaf RWC other mechanisms are likely to be involved.

### 4.2. Accumulation of sHSPs during Dehydration at Optimal and High Temperatures

The reduced photosynthetic activity and enhanced level of the non-photochemical quenching were accompanied by an increased accumulation of sHSPs. Many authors have shown a direct correlation between the accumulation of sHSPs and plant stress tolerance [19,21,24,48,49,50,51]. Upregulation of sHSPs gene expression and accumulation of these proteins under stress conditions suggest their importance in acquiring/conferring tolerance to different environmental conditions, especially to high temperature [19,52,53]. In the present study, we showed for the first time the protein expression profile of sHSPs in *H. rhodopensis* plants during desiccation at high temperature, which is common in natural environments. Drought stress induced the expression of only two isoforms of sHSPs at 50% RWC. Our results clearly showed high temperature dependent accumulation of three sHSPs also in control plants, kept at 38 °C (C3d). It is interesting to note that after three days at 38 °C (C3d) control plants accumulated sHSPs in larger quantities compared to controls kept at high temperature for seven days (C7d) as was visible from the immunoblot signals after 1D SDS-PAGE. Surprisingly, under dehydration at high temperature at 20% RWC, fourteen sHSP isoforms were present, five of them been different compared to those in leaves of air-dried plants. One day rehydration (R1) of dry plants at 38 °C led to a sharp decline of sHSPs protein expression in *H. rhodopensis*, unlike in fully recovered plants (R7), where the levels of sHSPs polypeptides were higher compared to R1, but below the levels of dehydrated samples. Decreased levels of sHSPs transcript during rehydration were observed also in other resurrection plants [31,34,35].

sHSPs were scarcely investigated in resurrection plants. The upregulation of sHSPs transcripts during the dehydration of resurrection plants *Boea hygrometrica*, *H. rhodopensis*, and *Reaumuria soongorica*, a resurrection semi-shrub, has been reported [31,32,33,34]. The accumulation of heat shock transcription factors (HSFs) during water stress of *B. hygrometrica* and *H. rhodopensis* also were detected [32,54]. Overexpression of heat shock transcription factor, *BhHsf1*, from *B. hygrometrica* in *Arabidopsis thaliana* and tobacco plants conferred increased thermotolerance of both plants [30]. Zhang et al. [31] identified 25 sHSPs genes in *B. hygrometrica* and they were differentially expressed depending on the stress conditions applied (dehydration, heat, cold, alkaline, high calcium, oxidation or application of ABA).

Our results showed the expression of different sHSPs with very close molecular weights (16.5–19 kDa) during desiccation at 23 °C and 38 °C. The newly synthesized sHSPs also differed in their pI values (5.0–8.0). In *B. hygrometrica*, six of ten cloned sHSPs exhibited acidic pI values (5.71–6.21), and another four, alkaline pI values (7.73–9.30) [31]. Differences in the pIs of sHSPs with the same molecular weight might be due to post-translational modifications of the proteins, with phosphorylation being the most common. The important role of protein phosphorylation in resurrection plants has been suggested [55,56].

The only data available on the protein expression of sHSPs in resurrection plants were provided by Alamillo et al. [35]. sHSPs accumulated after high temperature treatment but were also constitutively expressed in untreated controls and after dehydration. The authors suggested that sHSPs were not sufficient to provide/ensure desiccation tolerance but contributed to the survival of plants during desiccation by protecting and/or restoring damaged cellular components due to their action as molecular chaperones (Alamillo et al., 1995). sHSPs transcripts in control leaves were detected also in *B. hydrometrica* and *H. rhodopensis* [31,32]. No correlation between transcript and protein levels of sHSPs was detected and this could be due to post-transcriptional and post-translational modifications [51,57]. However, our results did not confirm a constitutive expression of sHSPs in control plants but confirmed the results of Rizsky et al. [58], showing that drought stress caused relatively small changes in the expression of sHSPs. It has been reported that only drought stress under high-light conditions induced the expression of sHSPs, but not under low-light conditions [59].

The physiological role of sHSPs upon dehydration at high temperature is still unclear [60]. Our previous results on the desiccation of *H. rhodopensis* at 38 °C showed that O_2_ evolution was very sensitive to heat stress. The recovery of the processes was very slow compared to the photochemical reaction, but nevertheless plants recovered their physiological functions [10,12]. The accumulation of the largest amount of sHSPs proteins in the late stages of desiccation of *H. rhodopensis* at 38 °C, when O_2_ evolution was very limited or not detectable, implied that they could have some role in thylakoid protection. sHSPs are thought to protect, but not repair, the electronic transport in mitochondria (complex I) and chloroplasts (oxygen-evolving complex) from the damaging effect of heat stress [59,61,62]. The thylakoid membrane stabilizing function of sHSPs was also suggested [63].

## 5. Conclusions

It should be emphasized that newly synthesized sHSPs in the late stages of dehydration (20%, 8% RWC) are most interesting in revealing the molecular basis of desiccation tolerance or thermotolerance, because such changes in expression are unique to resurrection plants, since drought-sensitive species do not withstand such low water content. Our future studies are envisaged to identify the newly discovered sHSPs using mass spectroscopy and to determine their role in *H. rhodopensis* resistance to high temperature and drought stress with the possibility to use them as markers for tolerance.

## Figures and Tables

**Table 1:** Different sHSPs isoforms accumulated in response to dehydration (D), high temperature (H) or dehydration at high temperature (D + H).

Specificity	Protein, No.
Induced by dehydration (D)	2, 9
Induced by high temperature (H)	3, 4, 5
Induced by dehydration at 38 °C (D + H)	1, 6, 7, 8, 10–14

**Table 2:** Molecular weight (kDa) of sHSPs detected by Western blot after 2D SDS-PAGE.

kDa	Protein, No.
19.0	1, 2
18.5	3, 4, 5, 10
18.0	6, 7, 12, 13
17.5	14
17.0	8, 9
16.5	11

## Data Availability

All datasets are contained within the article.
